# Supplementary material for: Calcium phosphate nanoclusters modify periodontium remodeling and minimize orthodontic relapse
Source: Biomaterials. Author manuscript; Available in PMC 2026 Aug 16. (PMC13477799; doi:10.1016/j.biomaterials.2024.122965)
Supplement: Supplementary file 1 [file NIHMS2191388-supplement-Supplemental.docx]

| **Material/Chemical** | **Vendor (Product/Catalog number)** |
| --- | --- |
| Calcium Chloride anhydrous | Bioworld (40300012-1) |
| Dibasic Sodium Phosphate; Dodecahydrate | Spectrum Chemical (S2056) |
| Poly(L-Aspartic Acid Sodium Salt); 14 kDa | Alamanda Polymers (X=100) |
| Poly (Acrylic Acid); 450 kDa | Polysciences (03312-100) |
| Sodium Hydroxide | Sigma Aldrich (71690) |
| Type 1- Insoluble Collagen | Advanced Biomatrix (5162) |
| Alizarin Red-S | Sigma Aldrich (A5533) |
| Mineral Oil | Bio Rad (163-2129) |
| L-Ascorbic Acid | Sigma-Aldrich (A4403) |
| Fetal Bovine Serum | Gemini Bio (100-106) |
| Penicillin/Streptomycin | Cytiva (Sv30010) |
| Phosphate Buffer Saline (pH 7.4) | Cytiva (Sh30256.01) |
| Paraformaldehyde (37%) | Spectrum Chemical (F0110) |
| 12 mm Nickel Titanium closed-coil spring | American Orthodontic (855-181) |
| 0.010“ stainless steel ligature | Ortho Technology (905-100) |
| Flowable Composite | Patterson dental (07-5020433) |
| Self-etch Adhesive Applicators | Patterson dental (07-5823018) |
| Buprenorphine | Patterson dental supply inc. (07-8925235) |
| Vinyl polysiloxane – Light Body Fast Set | Patterson dental supply inc. (07-6761440) |
| Yellow model stone | Patterson dental supply inc. (07-4265435) |
| Formalin (10%) | Fisher Scientific (22-110-758) |
| Ethanol (200 proof) | VWR (TX89125-172SFU) |
| Ethylenediaminetetraacetic (EDTA) | Research Products International (E57045-500.0) |
| Direct red 80 (Sirius red) | Sigma-Aldrich (365548) |
| Trichrome stain (Masson) kit | Sigma-Aldrich (HT15) |
| Harris Hematoxylin | VWR (95057-858) |
| Eosin | VWR (95057-848) |
| Differentiation solution | Sigma-Aldrich (A3179) |
| Type-I collagen (rat tail) | Gibco (A10483-01) |
| SafeClear II (Xylene Substitute) | Fisher (23044192) |

**Supplemental table 1:** List of Chemicals and materials used in this study

**Supplemental Table 2. Summary of μCT quantifications.** ^a^ p<0.05 vs baseline; ^b^ p<0.05 vs baseline; ^c^ p<0.05: PBS vs HC-PILP

**Supplemental Figure 1.** Transmission electron micrographs of as-prepared PAA/PASP alone (left) and HC-PILP (right) with selected area electron diffraction (SAED) insets. Scale bar = 50 nm.

**Supplemental Figure 2.** (**A**) Transmission electron micrograph of hydroxyapatite crystal with selected area electron diffraction (SAED) (inset). (**B**) X-ray diffraction of hydroxyapatite (red) and corresponding peak fitting profile (blue; ICDD card number: *01-071-6621*).

**Supplemental Figure 3.** X-ray diffraction of HC-PILP after reactant mixture prior to pH adjustment (green), after pH-adjustment (blue), 1 hour after synthesis (pink), and polymer only control (black). (**B-E**) Elemental Dispersive X-ray spectroscopy of collagen type-I fibers after incubation with HC-PILP for 7 days. (**F**) Collagen maturity represented as the ratio of 1660/1690 peak heights from FTIR spectra. **(G-I**) (**A**) Bar graph of gene expression data from MC3T3-E1 after 14D of stimulation with PAA/PASP and HC-PILP.

**Supplemental Figure 4.** Transmission electron micrographs of collagen Type-I fibrils after incubation with water (H_2_O) (**A**), PAA/PASP(**B**), or HC-PILP (**C**) for 7 days. Scale bar = 1 μm.

**Supplemental Figure 5.** (**A**) Animal weight (grams) throughout the duration of the experiment. p>0.05 determined by a Two-way ANOVA indicating that the weight of the animal was not affected throughout the 56 -day experiment. A gradual increase in weight is observed after the injection of the experimental groups indicating that they did not influence the eating habits of the animals. **(B-F**) microCT 3D reconstructions of hemi maxilla from baseline (B) and post-orthodontic tooth movement controls (C) as well as after 24 days with treatment with PBS (D), PAA/PASP (E), or HC-PILP (F). Red dashes line outlines the PDL space.

**Supplemental Figure 6. (A)** Picrosirius polarized light microscopy (4x) of the interradicular bone and 5 roots of the 1^st^ molar (Mesial root (MR), Buccal Root (BR), Distal Buccal Root (DBR), Mesial Lingual Root (MLR), and Distal Lingual Root (MLR)). Blue box depicts the mesial surface of the DBR and the surface undergoing compression during orthodontic tooth movement. Grey box depicts the distal surface of the MR and the surface undergoing tension during orthodontic tooth movement. Scale bar 500 μM. **(B)** Higher magnification (20x) of colored boxes with outlined regions of collagen detected above threshold (5/256). Scale bar = 200 μM.

**Supplemental Figure 7.**  Masson Trichrome micrographs (20x) of at the mesial surface of the distal buccal root PBS, PAA/PASP, and HC-PILP treated groups at 12- and 24-days relapse. Scale bar = 100 μM.

 **Supplemental Figure 8.** Hematoxylin and Eosin (H&E) micrographs (40x) at the mesial surface of the distal buccal root (**A)** and the distal surface of the mesial root (**B**) of baseline and post-orthodontic tooth movement (Post-OTM) controls as well as PBS, PAA/PASP, and HC-PILP treated groups at 4-, 12-, and 24-days relapse. Scale bar = 50 μM.

 **Supplemental Figure 9.**  Masson Trichrome micrographs (20x) at the distal surface of the mesial root of baseline and post-orthodontic tooth movement (Post-OTM) controls as well as PBS, PAA/PASP, and HC-PILP treated groups at 4-, 12-, and 24-days relapse. Scale bar = 100 μM.

 **Supplemental Figure 10.** (**A-K**) Picrosirius polarized light microscopy at the distal surface of the mesial root. Representative images are shown after treatment with PBS (**C-E**), PAA/PASP (**F-H**), and HC-PILP nanoclusters **(I-K**) after 4, 12, and 24 days of relapse. Baseline and Post-OTM are shown for comparison. White dashes outline indicates bone and tooth borders. Scale bar = 50 μM. Bar graph of collagen detection across different color channels: Red **(L**) and Green **(M**). p<0.05 determined by One-way ANOVA with a Tukey’s post-hoc test for multiple comparisons (n=3-6, per group at each time point). All data is represented as mean and standard deviation.

**R24**

 **Supplemental Figure 11.** Bar graph of gene expression data from NIH-3T3 fibroblast after stimulation with Transforming Growth Factor Beta (TGF-β) for 48 hours.

p<0.05 determined by One-way ANOVA with a Tukey’s post-hoc test for multiple comparisons (n=4). All data is represented as mean and standard deviation.

 **Supplemental Figure 12.** **(A**) Collagen turbidity (orange) shows an increase in 405 nm absorbance indicative of collagen fibrillogenesis. No signal is observed from the water (light blue), PBS (green), HC-PILP in water (purple), or PAA/PASP in water (blue) controls, which indicates no interference signal to detect collagen turbidity. (**B-C**) Collagen turbidity is dose-dependent with HC-PILP nanoclusters and PAA/PASP alone. Dose concentrations are in Table 1 of the main manuscript.

**Supplemental Figure 13.** Transmission electron micrographs of collagen Type-I after fibrillogenesis and incubation with low and high doses of PAA/PASP and HC-PILP. Assay was performed according to the collagen turbidity assay, negative stained, and imaged. Scale bar = 2 μM.
